# Symptom clusters after chemoradiotherapy in discharged nasopharyngeal carcinoma patients

**DOI:** 10.3389/fonc.2023.920889

**Published:** 2023-06-14

**Authors:** Xiang Li, Gui-Fen Fu, Yu-Ying Huang, Qing-Yu Jiang, Xiao-Yan Huang, Jin-Tao Zhang, Shen-Hong Qu

**Affiliations:** ^1^ Department of Otorhinolaryngology, The People's Hospital of Guangxi Zhuang Autonomous Region, Guangxi Academy of Medical Sciences, Nanning, China; ^2^ Department of Nursing, The People's Hospital of Guangxi Zhuang Autonomous Region, Guangxi Academy of Medical Sciences, Nanning, China

**Keywords:** nasopharyngeal carcinoma, chemoradiotherapy, symptom clusters, nursing, otorhinolaryngology

## Abstract

**Objective:**

To investigate the incidence of complications and types of chemoradiotherepy induces symptom clusters in patients with nasopharyngeal carcinoma (NPC) who were first diagnosed after treatment and discharged from hospital.

**Methods:**

After their discharge home, 130 NPC patients who had been treated with chemoradiotherapy were asked to complete a modified Chinese version of the *Quality of Life Questionnaire–Head and Neck Module* developed by the European Organization for the Research and Treatment of Cancer in the Head and Neck. Symptom clusters in patients were identified through exploratory factor analysis.

**Results:**

The most serious symptoms for discharged NPC patients who had received chemoradiotherapy were dental problems, a sense of obstruction while swallowing, embarrassment in physical contact with family members and friends, difficulty in speaking with others, and embarrassment in public. The six symptom clusters identified through exploratory factor analysis were (1) painful eating, (2) social difficulties, (3) psychological disorders, (4) symptomatic shame, (5) teeth/throat injuries, and (6) sensory abnormalities. The total contribution rate of variance was 65.73%.

**Conclusion:**

NPC patients who are treated with chemoradiotherapy can experience adverse symptom clusters that continue after discharge. Nurses should evaluate the patients’ symptoms before discharge and provide targeted health education services which would reduce the patients’ complications and improve the quality of life at home. Besides, medical staff should evaluate the complications in a timely and comprehensive manner and provide individualized health education for the affected patients to help them manage chemoradiotherapy side effects.

## 1 Introduction

Nasopharyngeal carcinoma (NPC) is the primary malignant tumor of the head, neck, and the epithelium that covers the nasopharyngeal mucosa. It is reported that the incidence of NPC is 1 in 100,000 (1). In China, 80% of NPC occurs in Guangdong, Guangxi, and other locations in South China; 92% of the newly-diagnosed cases are also located in these areas (2). Radiotherapy combined with adjuvant chemotherapy is the primary therapeutic method for NPC. The side effects of chemoradiotherapy for NPC are including early and long-term effects (3). Early side effects present during the process of therapy and during the immediate post therapy period, mucositis is the most common early side-effect to the oral mucosa with severe pain, impaired swallowing and malnutrition (4, 5). Late effects can occur from weeks to several years after its completion including xerostomia and dysgeusia, hearing loss, consistent xerostomia, mandibular osteoradionecrosis and dysphagia (6, 7). Serious complications may interrupt the treatment, affect the therapeutic results, shorten the interval of tumor recurrence, and affect the patients’ quality of life.

The complications caused by chemotherapy occur in the form of symptom clusters that severely affect the quality of life of NPC patients. Liao et al. (8) found that patients discharged after NPC chemoradiotherapy asked for further health education. The patient’s discharge might be delayed for days or even weeks due to serious complications such as myelosuppression and gastrointestinal reactions. The medical staff should be aware of the patients’ complications before the end of treatment so that the patients may undertake effective self-care as they return to their homes, families, and social roles. This study will provide a theoretical basis for the clinical management of cluster symptoms resulting from chemoradiotherapy side effects in NPC patients.

## 2 Subjects and methods

### 2.1 Research subjects

Using a convenience sampling method, 130 patients with NPC were enrolled in the study. They had been treated in The People's Hospital of Guangxi Zhuang Autonomous Region, Guangxi Academy of Medical Sciences from April to November 2017. The authors interviewed NPC patients 48 hours after hospital discharge to assess their symptom clusters. The patients had received the same therapeutic protocols:

Radiotherapy: A linear accelerator was used for intensity-modulated radiotherapy with an average dose of 72Gy/7~8W.

Chemotherapy: Two cycles of chemotherapy with platinum + 5-fluorouracil were administered. A micropump continuously injected 5-fluorouracil for 120 hours and once every 28 days. The regime of chemotherapy was cisplatin (30 mg/m2 per day for 3 days)with 5-fluorouracil (2000 mg/m2 for 5 days) in this cohort.

Patients were selected for the study using the following criteria:

Inclusion criteria: (1) first-time NPC patients diagnosed by histopathology who underwent chemoradiotherapy, (2) no additional malignant tumors, (3) conscious and able to communicate normally. (4) The Karnofsky Performance Status (KPS) of all patients recruited were more than 80.

Exclusion criteria: (1) age less than 18 years old, (2) hearing impairments, and (3) deceased or unable to complete the study for other reasons.

### 2.2 Investigation methods

The authors interviewed NPC patients 48 hours within hospital discharge to assess their symptom clusters. After unified training, questionnaires were distributed by two research nurses with master’s degree. The questionnaires were completed face-to-face with the patients, 48 hours before discharge, and collected. At the beginning of the survey, the purpose of the study was explained, and each item was clarified to ensure full understanding by the respondent. If the patient had a vision impairment or physical limitations, the investigators read the questions. A total of 130 questionnaires were issued and 130 valid questionnaires were returned. The effective recovery was 100%.

#### 2.2.1 Investigation tools

##### 2.2.1.1 General data

The questionnaire included demographic data (gender, age, nationality, occupation, educational background, and marital status) and disease-related information (body mass index, smoking and drinking history, therapeutic protocols, and pathological staging).

##### 2.2.1.2 Research data

The researchers used a modified Chinese version of the *Quality of Life Questionnaire–Head and Neck Module*, developed by the European Organization for the Research and Treatment of Cancer in the Head and Neck (EORTC QLQ-H&N35).

The 35 questions include seven symptomatic domains (head and neck pain, swallowing function, sense, speech, social eating, social contact, and sexuality) and eleven single-symptom items (dental effects, mouth movements, dry mouth, sticky saliva, coughing, ill-feeling, use of pain killer, nutritional supplements, feeding tube, weight gain, and weight loss). Items 1 to 30 are scored from 1 to 4 according to four Likert’s grades: “no,” “a little,” “equivalent,” and “unusual.” Items 31 to 35 are scored “yes” or “no.” A higher score equals more severity in the symptom area and a diminished quality of life. The Cronbach’s coefficient α of the scale was 0.623~0.970, indicating good reliability and validity (9). This study used the first 30 symptoms of the QLQ-H&N35 to extract the symptom clusters.

#### 2.2.2 Statistical methods

SPSS20.0 software was used for statistical analysis. The general characteristics of the patients were labeled by the descriptive statistics, and the counting data were described by frequency, ratio, or percentage. Measurement data subject to a normal distribution used means ± standard deviation. For measurement data not subject to normal distribution, *M*(*P_25_, P_75_*) was adopted in the statistical description. Exploratory factor analysis was used for the symptom clusters.

## 3 Results

### 3.1 General characteristics of patients with NPC

The general characteristics of the NPC patients are presented in **Table 1**.

**Table 1:** The general characteristics of patients with NPC (*n*=130).

Projects	Cases	Percentage (%)	Projects	Cases	Percentage (%)
Gender			Payment method		
Male	91	70	Health care	128	98.5
Female	39	30	At his own expense	2	1.5
Age(years)			Body mass index (bmi)		
<60	111	85.4	<18.5	13	10
≥60	19	14.6	18.5~	84	64.6
Nation			≥25	33	25.4
Han Nationality	49	37.7	Smoking		
zhuang	75	57.7	Yes	36	27.7
Others	6	0.6	No	94	72.3
Occupation			Drinking		
Farmers	41	31.5	Yes	23	17.7
Staff	22	16.9	No	107	82.3
Workers	22	16.9	Treatment options		
Others	45	34.7	Radiotherapy plus chemotherapy	127	97.7
Educational level			Radiation therapy	1	0.8
Junior high school and below	95	73.0	Surgery plus chemoradiotherapy	2	1.5
High School and Technical Secondary School	18	13.8	The pathological staging		
Junior college or above	17	13.2	I	5	3.9
Marital status			II	16	12.3
Unmarried	10	7.7	III	58	44.6
Married	112	86.2	IV	51	39.2
Divorced or widowed	8	6.1			

### 3.2 The incidence and severity of symptoms in NPC patients at the end of treatment

At the end of treatment, patients had the highest incidence in eight of the 30 QLQ-H&N35 symptoms: dry mouth (98.5%), difficulty in swallowing (96.9%), sticky saliva (96.9%), aversion to eating (96.9%), abnormal taste (96.2%), sore throat (93.1%), ill-feeling (92.3%), and difficulty in eating (86.9%). The symptoms with the highest severity and their *M*(P_25_,P_75_) scores were dental problems: 94.5 (32.0, 94.5); a feeling of obstruction while swallowing: 92.0 (44.5, 92.0); embarrassment during physical contact with family and friends: 89.0 (30.0, 89.0); difficulty in speaking: 87.5 (31.5, 87.5); and embarrassment in public: 87.5 (27.0, 87.5) (**Table 2**).

**Table 2:** The incidence of complications and the severity after the completion of treatment in patients with NPC (*n*=130).

Symptoms	Occurrence rate [Cases(Percentage,%)]	Score for severity [Score, *M*(P_25_,P_75_)]	Symptoms	Occurrence rate [Cases(Percentage,%)]	Score for severity [Score, *M*(P_25_,P_75_)]
Oral Pain	81(62.3)	70.0(25.0,10.5)	Hoarse voice	95(73.1)	75.5(18.0,75.5)
Pain in the jaw	57(43.8)	37.0(37.0,96.0)	Ill-feeling	120(92.3)	83.0(27.5,83.0)
Sharp pain in the mouth (ulcer)	99(76.2)	49.5(49.5,91.0)	Worrying about a change in appearance	78(60.0)	82.5(26.5,82.5)
Sore throat	121(93.1)	68.5(25.5,113)	Difficulty in eating	113(86.9)	67.5(26.0,67.5)
Difficulty in drinking slop	94(72.3)	58.5(18.5,97.5)	Embarrassing to eat in front of your family	15(11.5)	58.0(58.0,58.0)
Difficulty in swallowing semi-liquid food	107(82.3)	49.5(49.5,94.0)	Embarrassing to eat in front of other people	19(14.6)	56.0(56.0,56.0)
Difficulty in swallowing solid foods	126(96.9)	82.5(24.0,82.5)	Aversion to eating	126(96.9)	62.0(62.0,112.5)
A feeling of obstruction while swallowing	103(79.2)	92.0(44.5,92.0)	Difficulty in speaking	68(52.3)	87.5(31.5,87.5)
Dental problems	67(51.5)	94.5(32.0,94.5)	Difficulty in talking on the phone with others	69(53.1)	86.5(31.0,86.5)
Difficulty in opening mouth	87(66.9)	79.0(22.0,79.0)	Difficulty in interacting with family members	64(49.2)	33.5(33.5,92.5)
Dry mouth	128(98.5)	52.5(52.5,109.5)	Difficulty in interacting with friends	81(62.3)	80.0(25.0,80.0)
Sticky saliva	126(96.9)	56.5(56.5,111.0)	Embarrassment in public	77(59.2)	87.5(27.0,87.5)
Dysosmia	49(37.7)	41.0(41.0,94.0)	A feeling of obstruction while swallowing	71(54.6)	89.0(30.0,89.0)
Abnormal taste	125(96.2)	65.0(24.5,108.5)	Feeling loss of Libido	85(65.4)	73.5(23.0,73.5)
Cough	95(73.1)	72.5(18.0,72.5)	Feeling less sexual pleasure	85(65.4)	73.0(23.0,73.0)

### 3.3 Analysis of the symptom clusters in NPC patients at the end of treatment

The calculation of the exploratory factor analysis was performed using the principal component analysis and maximum variance rotation. In the Kaiser-Meyer-Olkin test, KMO = 0.829. The Bartlett sphericity test of P < 0.001 suggested that the data in the present study were suitable for factor analysis. Six factors with an eigenvalue > 1.3 were extracted for analysis. The total contribution rate of variance was 65.73%. The symptoms with a factor load > 0.6 were included in the symptom clusters. The six symptom clusters had high internal reliability, and the Cronbach’s α coefficients were 0.903, 0.937, 0.881, 0.969, 0.609, and 0.497, respectively. The load component matrix of the symptom cluster factors after rotation is shown in **Table 3**.

**Table 3:** Analysis of symptom clusters in admitted patients with NPC after the completion of treatment (*n*=130).

Symptoms	Factor load					
	Factor 1	Factor 2	Factor 3	Factor 4	Factor 5	Factor 6
Oral Pain	-0.143	-0.059	0.221	0.085	-0.202	0.015
Pain in the jaw	0.201	0.241	0.020	0.346	0.194	-0.004
Sharp pain in the mouth (ulcer)	0.433	0.497	-0.126	0.140	0.256	0.179
Sore throat	0.755	0.357	0.003	0.087	0.093	-0.123
Difficulty in drinking slop	0.778	0.328	0.196	0.203	0.026	-0.138
Difficulty in swallowing semi-liquid food	0.790	0.239	0.283	0.150	0.024	-0.122
Difficulty in swallowing solid foods	0.773	0.002	0.068	-0.100	0.064	0.324
A feeling of obstruction while swallowing	0.576	0.010	0.137	0.150	0.505	0.096
Dental problems	0.081	-0.100	0.056	0.197	0.675	0.163
Difficulty in opening mouth	0.198	0.279	0.047	0.238	0.307	-0.021
Dry mouth	0.441	0.067	0.122	-0.188	0.003	0.144
Sticky saliva	0.542	0.101	0.177	-0.174	0.221	0.158
Dysosmia	0.083	0.206	0.016	0.331	-0.247	0.618
Abnormal taste	0.059	0.156	-0.040	-0.126	0.209	0.821
Cough	0.147	0.269	0.204	-0.100	0.684	-0.236
Hoarse voice	0.146	0.253	0.192	-0.206	0.515	0.170
Ill-feeling	0.533	0.245	0.294	-0.124	0.204	0.217
Worrying about a change in appearance	0.175	0.022	0.747	0.093	0.214	-0.083
Ddifficulty in eating	0.755	0.180	0.198	-0.005	0.131	0.268
Embarrassing to eat in front of your family	0.035	0.204	0.303	0.840	0.106	-0.055
Embarrassing to eat in front of other people	0.031	0.249	0.301	0.845	-0.016	-0.008
Aversion to eating	0.451	0.287	0.051	-0.100	0.282	0.553
Difficulty in speaking	0.210	0.850	0.162	0.224	0.121	0.118
Difficulty in talking on the phone with others	0.216	0.850	0.192	0.251	0.111	0.106
Difficulty in interacting with family members	0.212	0.882	0.184	0.099	0.013	0.077
Difficulty in interacting with friends	0.196	0.730	0.311	0.000	-0.060	0.209
Embarrassment in public	0.135	0.242	0.775	0.256	0.117	0.123
A feeling of obstruction while swallowing	0.252	0.307	0.732	-0.042	0.170	-0.087
Feeling loss of Libido	0.103	0.139	0.763	0.207	-0.028	0.042
Feeling less sexual pleasure	0.102	0.137	0.763	0.235	-0.043	0.034
Cronbach’α coefficient	0.903	0.937	0.881	0.969	0.609	0.497

According to the factor load after rotation, the following six symptom clusters were evident in the present study: Factor 1: painful eating and swallowing (sore throat, difficulty in drinking liquid, difficulty in swallowing semiliquid food, and difficulty in swallowing solid food); Factor 2: social anxiety difficulties (speaking with others, speaking on the phone, and socializing with friends and family members); Factor 3: psychological disorders (worried about changes in appearance, embarrassed to appear in public, embarrassed to have physical contact with family members and friends, decreased sexual desire, and decreased pleasure in sexuality); Factor 4: symptomatic shame (embarrassment while eating in front of other people and shame from being a cancer patient); Factor 5: teeth/throat injuries (dental problems and cough); and Factor 6: sensory abnormality (abnormal taste and smell).

## 4 Discussion

### 4.1 Frequent adverse symptoms of NPC patients after treatment: dental disease, swallowing obstruction, and social anxiety

At present, chemoradiotherapy is still the primary therapeutic protocol for patients with NPC. The side effects of chemoradiotherapy are present throughout the entire treatment process. Serious complications may interrupt the treatment, prolong the length of hospital stay, and reduce the quality of life. After completion of the therapy, the side effects of chemoradiotherapy continue after the patients return home. The medical staff should evaluate the complications before discharge and provide an individualized education plan for each patient.

The quality of life of the NPC patients was measured using the EORTC QLQ-H&N35, and 25 of the 30 symptoms had an incidence of more than 50%, meaning that the patients suffered from a variety of symptoms. Among these symptoms, the most serious were dental problems (*M*[P_25_,P_75_] = 94.5 [32.0, 94.5]), followed by a feeling of obstructed swallowing (*M*[P_25_,P_75_] = 92.0 [44.5, 92.0]). In previous studies, Sroussi et al. found that the most serious complications of head and neck radiotherapy were oral mucositis, dry mouth, and malnutrition (10). Myelosuppression is one of the most common complications of chemoradiotherapy, which indicates that the immune function of the body is damaged and has decreased immunity (11).

Radiation can destroy the periodontal connective tissue, causing injury to the periodontal mucosa defense system and resulting in gum swelling and pain, oral ulcers, oral microflora, other oral microenvironment disorders, and oral infection (11), especially *Candida albicans* infection (12, 13). Schuurhuis et al. (14) found that patients who received intensity-modulated radiotherapy and concurrent chemoradiotherapy had an increase in oral *Escherichia coli*, *Staphylococcus*, *Candida*, and other opportunistic pathogens. Radiation has a direct effect on teeth, and the atrophy of gingiva and interdental papilla results in the exposure of tooth roots, food impaction and bacterial retention, a decrease in saliva secretion, a decrease in oral self-cleaning ability, and radiation caries (15). Although the patients had teeth cleanings and periodontal disease prevention before the chemoradiotherapy treatment, they were not examined during the treatment period, and dental problems developed.

After discharge, NPC patients returned to a wider social network and often experienced social anxiety symptoms. Li et al. found that patients with NPC generally had emotional problems and psychological pain, often brought on by their physical challenges (16).

More than 95% of the patients in our study experienced dry mouth, sticky saliva, abnormal taste, difficulty in swallowing solid food, and reduced enjoyment from food. Although these difficulties are less consequential than dental disease, swallowing obstruction, or social disorder, they seriously affected the quality of life at home and should be brought to the attention of medical staff during follow-up.

### 4.2 Patients with NPC experienced six symptom clusters after treatment

As described above, the six symptom clusters were (1) painful eating and swallowing, (2) social anxiety, (3) psychological disorders, (4) symptomatic shame, (5) teeth/throat injuries, and (6) sensory abnormalities.

#### 4.2.1 Painful eating and swallowing

In the present study, the symptom cluster of painful eating and swallowing included sore throat, difficulty in drinking liquid, difficulty in swallowing semiliquid food, difficulty in swallowing solid food, and difficulty in eating. Shen et al. reported that patients in the late stage of radiotherapy treatment suffered from sticky saliva, dysphagia, taste abnormality, dry mouth, and oral/throat pain (17).

These previously reported symptoms were not entirely consistent with the findings of the present study, which might be due to different symptom evaluation scales and statistical methods. Almost all patients with NPC who are undergoing chemoradiotherapy will have radiation stomatitis and laryngopharyngitis (18); as the doses increase, patients are often unable to swallow at will because of the severe pain.

At the same time, radiotherapy may cause fibrosis of the muscles involved with swallowing through extracellular matrix deposits and fibroblast proliferation. Radiotherapy damages up to 30 pairs of muscles and six pairs of cranial nerves (19), leading to a loss of muscular strength, coordination, swallowing function, and eventually dysphagia. Because the salivary glands might be injured by the radiation, the secretion of saliva is reduced, which also impairs the swallowing process. Patients also suffer from dysphagia due to acute oropharyngeal cavity pain, which should be managed in clinical practice to ensure that they eat sufficiently and receive adequate daily nutrition.

#### 4.2.2 Social anxiety

The social anxiety symptom cluster included difficulties in speaking with others, speaking on the phone, and socializing with friends and family members. The negative emotions of patients often come from a lack of awareness and understanding of radiation therapy, as well as the heavy economic burden. In addition, the deeper the treatment, the more obvious the toxic and side effects brought by radiotherapy and chemotherapy. Patients who are unable to bear the toxic and side effects are prone to develop anxiety, panic, suspicion, and even give up treatment, ultimately affecting the effectiveness of treatment and rehabilitation.

It is reported that 60.62% of patients with head and neck cancer have depressive symptoms (20). The majority of patients with NPC are young and middle-aged males, who are often the breadwinners for their families and are in the prime of their careers and personal lives. The occurrence of tumors and the side effects of chemoradiotherapy may cause different degrees of physical and mental distress to the patients and make them reluctant to communicate with their loved ones. The patients also fear letting others know about their illness because it may negatively affect their future opportunities.

These negative physical and mental problems will seriously harm the quality of life of the NPC patients and may even cause a recurrence of the cancer. In addition, the incidence of cancer-related fatigue in patients with NPC is as high as 82.5%, which is inversely proportional to the amount of social support the patient does receive. Providing good social support can effectively improve the patients’ anxiety, depression, and quality of life (21). Noh et al. (22) reported that ratios of anxiety, substance abuse, and soma to form/conversion disorders fell after the start of radiotherapy compared to before radiotherapy.

#### 4.2.3 Psychological disorders

The psychological disorder symptom cluster consisted of worry about changes in appearance, embarrassment at appearing in public, embarrassment during physical contact with friends and family members, a decreased sex drive, and decreased pleasure in sexual activities. Radiotherapy to the head and face causes increased pigmentation, hair loss, nasal hemorrhage, and necrosis of the nasal tissue; these undesirable changes in appearance are a source of negative emotions. Decreased libido is a common complaint in cancer patients. However, another study (23) suggests that there is no significant difference in the sexual function in NPC patients between three months to one year after treatment, suggesting that sexual function might be correlated with adverse emotions such as anxiety and depression during the treatment.

#### 4.2.4 Symptoms of shame

The symptoms in the shame cluster range from common feelings such as embarrassment at eating in front of other people to more profound feelings of shame caused by being a cancer patient, which is manifested as guilt, shame, self-reproach, and fear of being discriminated against (24). At present, the majority of the research on symptomatic shame has focused on patients with lung and breast cancers (25), with less attention given to head and neck carcinomas. Chemoradiotherapy for NPC injures the head and face and can also lead to more serious complications such as deafness and blurred vision. Patients may suffer from dysphagia and radiation oropharyngeal mucositis, which can cause severe pain and slow the speed of eating. Most of the food taken by the patients is liquid or semiliquid, and their eating habits are different from those of healthy individuals. When patients return to their families, they may suffer from different degrees of symptomatic shame.

#### 4.2.5 Teeth/throat symptoms

The teeth/throat injury symptom cluster included dental problems and cough, which might be primarily caused by radiation injury. Radiation damages teeth through the destruction of the enamel surface layer, the formation of craters under the surface layer, the exposure of dentin under the surface layer, the formation of a wide range of porous structures, and the development of radioactive caries (26). Most of the NPC patients were affected by subjective discomforts such as tooth or gingival tenderness and tooth sensitivity. In addition, radiotherapy can cause dry mouth, sore throat, thick throat sputum, frequent coughing, and a decreased self-cleaning ability of the mouth, all of which might aggravate the symptoms of tooth-throat injury.

#### 4.2.6 Sensory abnormalities

The sensory abnormality symptom cluster included abnormal taste and smell. In the present study, most of the patients with NPC received one cycle of induction chemotherapy followed by radiotherapy, and the abnormal taste and smell often occurred in the radiotherapy stage. Most patients complained that the changes of taste resulted in a loss of appetite, a decrease in food intake, and changes in eating habits. This led to anxiety and depression and affected their quality of life (27, 28).

Shen et al. (17) extracted four symptom clusters from 133 NPC cases in the late radiotherapy stages of concurrent chemoradiotherapy: emotion-pain, diseased feeling, and digestive tract symptom clusters. Xiao et al. (29) extracted four symptom clusters: general, gastrointestinal, nutritional, and social. The findings of our study are consistent with the symptom clusters found in previous studies, except for the digestive tract symptom cluster. The difference might be caused by different symptom evaluation forms and different evaluation time points.

## 5 Limitations

This study was a single-center study, and the number of study population was relatively small. Thus, the results of this study need to be further confirmed by a larger study population in a multicenter. Besides, this study did not consider the physical performance of the patients before starting the chemoradiation, which might have an influence to the results of this study. In the management of the discharged NPCs, it should be careful about eating, social, psychological, symptomatic, and sensory condition, which is helpful to manage cluster symptoms resulting from chemoradiotherapy side effects in NPC patients. Finally, due to small sample size and incomplete information, this study did not analyze and compare the symptoms after chemoradiation in NPC patients between two criterias, for example, between man and woman, between early stage and advance stage, or between young and old patients. In the future, multi-center studies with large samples and at least 6 months of follow-up data should be carried out.

## 6 Conclusion

NPC patients are troubled by many symptom clusters, especially dental disease, social challenges, and a sense of obstruction while swallowing. Patients suffer a diminished quality of life and difficulty in returning to their homes because of a number of disorder symptom clusters: painful eating, social anxieties, psychological disorders, symptomatic shame, teeth/throat injuries, and sensory abnormalities. Nurses should evaluate the patients’ symptoms before discharge and provide targeted health education services. This will help to reduce the patients’ complications and improve the quality of life at home.

## Data availability statement

The original contributions presented in the study are included in the article/supplementary material. Further inquiries can be directed to the corresponding author.

## Ethics statement

The studies involving human participants were reviewed and approved by Ethics Committee of The People’s Hospital of Guangxi Zhuang Autonomous Region. The patients/participants provided their written informed consent to participate in this study.

## Author contributions

G-FF and XL conceived the idea and conceptualised the study. Y-YH and Q-YJ collected the data. X-YH and J-TZ analysed the data. XL and G-FF drafted the manuscript, then S-HQ reviewed the manuscript. All authors read and approved the final draft. All authors contributed to the article and approved the submitted version.
